# Hoop training: a pilot study assessing the effectiveness of a multisensory approach to treatment of body image disturbance in anorexia nervosa

**DOI:** 10.1007/s40519-018-0585-z

**Published:** 2018-10-04

**Authors:** Anouk Keizer, Manja M. Engel, José Bonekamp, Annemarie Van Elburg

**Affiliations:** 1 Faculty of Social and Behavioural Sciences, Experimental Psychology, Utrecht University, Heidelberglaan 1, 3584 CS Utrecht, The Netherlands; 2 Altrecht Center for Eating Disorders Rintveld, Altrecht Mental Health Institute, Wenshoek 4, 3705 WE Zeist, The Netherlands

**Keywords:** Anorexia nervosa, Eating disorders, Body image disturbance, Multisensory, Treatment

## Abstract

**Purpose:**

We tested in a pilot study a new intervention for body image disturbance in anorexia nervosa (AN). Unlike common treatment approaches our hoop training targeted not only cognitive-emotional and visual aspects of body image, but also tactile and body-scaled action components.

**Methods:**

We assessed cognitive, visual, tactile and body-scaled action aspects of body image disturbance before and after completion of hoop training. Twelve AN patients completed treatment as usual (TAU) for body image, 14 completed hoop training in addition to TAU.

**Results:**

Results show that patients who completed the 8-week individual hoop training in addition to TAU improved more on body image disturbance tasks from baseline to follow-up than patients who completed only TAU. Hoop training specifically seems to affect tactile body image and body-scaled action.

**Conclusions:**

Taken together, a treatment approach in which the full spectrum of body image disturbances in AN is targeted has a unique added effect over treatment as usual.

**Level of evidence:**

Level II, non-randomized controlled study.

## Introduction

Researchers and clinicians familiar with anorexia nervosa (AN) are likely to agree that body image disturbances are a very remarkable and persistent symptom of AN [1]. Despite being extremely thin, AN patients often perceive and experience their body shape and size in a distorted way, larger than it actually is. Body image disturbance plays a central role in AN and is linked to onset, maintenance, worse prognosis, decreased treatment success, and higher relapse rates [2].

Both literature and clinical experience show that it is difficult to successfully treat body image disturbances in AN [3]. Currently, body image disturbance is usually treated using some form of CBT and/or exposure therapy [4], in which the focus is almost exclusively on cognitive/emotional and/or visual aspects of body image disturbance. This is striking, as literature shows that body image disturbance in AN also expresses itself through other modalities (for overviews see e.g., [5, 6]), such as haptic/tactile processing, body-scaled action, and multisensory integration. These disturbances have been found even after recovery [3, 7]. Consequently, it may be argued that current treatment for body image disturbances in AN is not yet optimal, as it does not focus on the full range of multisensory disturbances in body size experience. Therefore a new therapeutic intervention for body image disturbance, targeting multiple modalities is presented in the current paper.

**Fig. 1:**
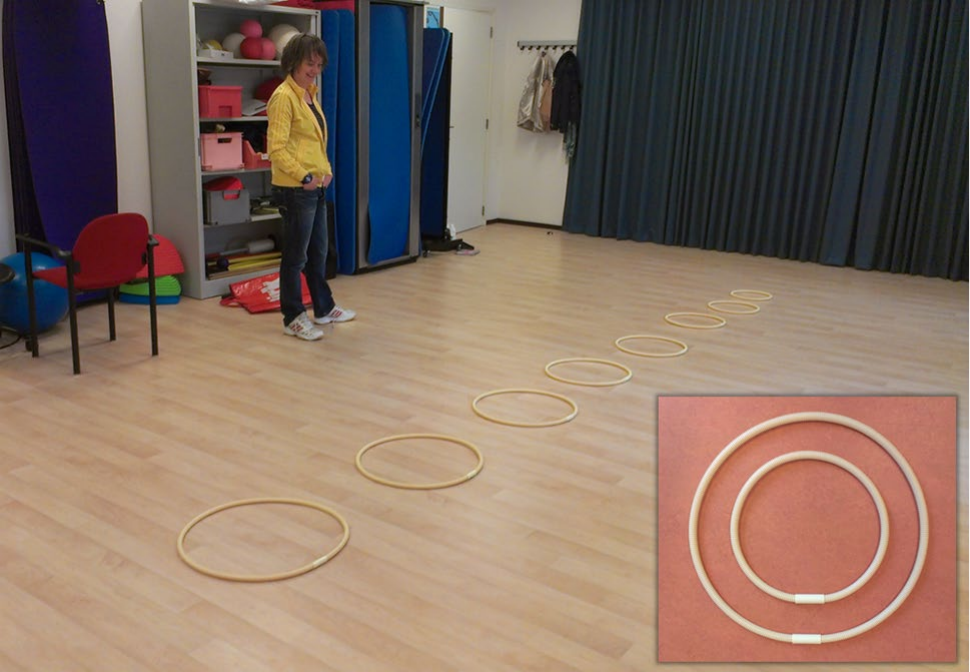
Setting in which hoop training takes place, the insert bottom right shows two hoops in close-up

This new intervention consists of an 8-week ‘hoop training’, offered to female AN patients once they are in a ± 4 kg range of their healthy target weight. The training consists of one session each week and is conducted by a psychomotor therapist. It takes place individually, with only the patient and therapist present. In each session eight hoops are lined up on the floor (see Fig. 1) and presented to a patient who stands in front of the hoops. These eight hoops are randomly selected out of a total of 15 hoops. The patient is thus unaware of the actual sizes of the hoops lying in front of her. By preventing her from knowing the exact sizes of the hoops, the patient is forced to rely on her internal model of body size, instead of using an external reference point (such as the diameter of the hoop in cm).

The insert at the right bottom of Fig. 1 shows a close-up of two different hoops. As can be seen the hoops look like traditional hula hoops. They are similar in weight (light, not comparable to fitness hula hoops), they just differ in size and production material. The hoops are made of flexible plastic tubing that is generally used to run electric cords through. Unlike traditional hula hoops, the hoops used in the training are flexible and can change shape from round to oval, which allows a patient to literally squeeze through the hoop if she wants. When standing in front of the eight hoops the patient is instructed to choose the one hoop that she perceives to fit her body exactly. After choosing a hoop the patient is asked to step inside that hoop and lift it up, over her head. The therapist has a coaching role, and stresses that the aim of the training is not to immediately select the best-fitting hoop, nor to be able to fit into the most-desired hoop, but to be able to practice with judging what the body can and cannot do. In this case, where it can and cannot fit through. In each session the patient is allowed to choose a hoop twice. This ensures that the patient can process the outcome of the first choice with guidance of the therapist, e.g., ‘was this hoop much too big for my body?’, reflect on it, e.g., ‘what can I learn from this?’, *and* take her first choice into account when choosing a hoop for the second time, e.g., ‘perhaps I should now choose a smaller hoop’.

Hoop training is different from other tactile and visual therapies that are in use today. First, a crucial aspect of hoop training is that patients actually move through the hoop of their choice, which gives them a very direct experience of their true body size. Besides creating a feedback loop for actual body size to the brain over the course of 8 weeks, hoop training is unique as it also targets other, *multisensory* aspects of body image. Thus, an advantage is that hoop training simultaneously activates different senses and aspects of body image: patients *see* the hoops lying in front of them, and have *thoughts*/*emotions* about them in relation to their body; also, they *feel* the hoop sliding over their body as they *move* through it. A key-factor of hoop training is that patients very directly experience that their body is smaller than they experience it to be as the size of the hoop is fixed and present during integration of the sensory feedback of the body and cognitions in the brain. When, e.g., drawing the own silhouette or when estimating the own waist circumference with a piece of rope, and comparing it to actual body size, the feedback is purely visual in nature. Similarly, during different touch or massage interventions the feedback is mainly tactile in nature, and often the therapist is the person who provides the tactile feedback. During hoop training the patient is fully in charge, she determines the hoops of her choice *and* she determines how she will deal with the outcome of her choice. For example, when during hoop training a patient is able to perform an action with her body, that she thought would not be possible (stepping through a small hoop), the feedback has a bigger impact, she cannot discard the action she has just performed and the fact that her *own* action offered proof indicating that she is thinner than she expected her body to be. In other words, she cannot “blame” someone or something else for fitting through a small hoop. When, e.g., outlining the body of a patient on the wall with a marker, a patient might look at it, reflect on it, and state something along the lines of “this silhouette looks thin, but you—the therapist—must have gone in too far when outlining my body, I am probably fatter than this outline”. In hoop training there is little room for such lines of reasoning, and as such, the eating disorder is given very little room. Moreover, with hoop training a climate is created in which a patient can experience success in terms of trusting her body and signals from different senses.

Given the wide range of currently available treatments for body image, hoop training was designed to have a duration per session of about 5–10 min, so that it can be easily added to TAU, or, if desired, used as a stand-alone treatment. Importantly, hoop training was not designed to replace existing treatments altogether, it is meant as an additional training next to existing therapies. Clinical experience indicates that in many existing interventions the feeling of fatness that patients experience is translated into underlying dysfunctional attitudes/emotions, and that disturbed *perception* of body size is not always taken into account. In others words, patients are provided with tools that teach them how to *cope* with attitudes/emotions related to ‘feeling fat’, while the occurrence of perceptually experiencing the body as fatter than it actually is, is not actively diminished. Learning to cope with attitudes/emotions associated with ‘feeling fat’ is useful and existing treatments are crucial for providing these coping strategies. The authors do however believe that hoop training has a unique added effect on top of TAU as it specifically aims to target body image disturbance as a whole, by focusing on attitudinal/emotional, visual, tactile, *and* movement aspects of body image in a single training.

In this pilot study one group of healthy participants and two AN groups were included. Healthy participants did not complete any training or treatment. One AN group completed TAU, the experimental AN group completed TAU *and* hoop training. It was expected that healthy participants would show no differences on any of the body image measures from baseline to follow-up. It was further hypothesized that improvement in the patients who completed body image TAU plus the hoop training would be more pronounced than in the patients who completed just body image TAU and not the hoop training.

## Methods

### Participants

Fifty females participated on the basis of written informed consent; 16 AN patients completed body image TAU plus the hoop intervention (AN_hoop), 14 AN patients completed only body image TAU (AN_tau), 20 healthy female controls (HC) received no intervention at all. Four participants did not complete the follow-up measures after 8 weeks (*N* = 1 AN_hoop; *N* = 2 AN_tau; *N* = 1 HC). These participants were removed from the data set and all subsequent analyses were performed on the remaining sample of 46 participants. Demographic information such as age and BMI can be found in Table 1.

**Table 1:** Clinical description of the sample

	AN_hoop (*N* = 14)		AN_tau (*N* = 12)		HC (*N* = 19)	
	*M*	SD	*M*	SD	*M*	SD
Age (years)	22.87	2.90	23.17	5.67	21.21	1.44
BMI at baseline^a^	19.53	1.04	20.11	1.17	20.80	1.61
BMI start treatment	16.77	2.43	17.09	2.46	n/a	n/a
Fat % start treatment	13.51	10.20	14.50	6.94	n/a	n/a
Shoulder width at baseline (cm)	40.63	2.25	40.00	2.38	41.47	1.98
Waist width at baseline (cm)	28.57	2.17	28.79	2.90	30.34	1.76
Hip width at baseline (cm)	34.47	1.26	35.63	2.92	37.50	1.99
Illness duration (months)^b^	7.00	3.93	12.29	10.32	n/a	n/a
Eating disorder inventory-II total score	252.20	31.23	251.58	25.74	153.63	31.20

^a^BMI is relatively high in the AN groups as treatment was offered to them when they were ~ 4 kg from their target weight

^b^Illness duration is defined as months since intake at the eating disorder facility

AN patients were recruited from Rintveld Centre for Eating Disorders, Altrecht Mental Health Institute in Zeist, The Netherlands. At this specialized eating disorder (ED) treatment center patients received treatment as usual ranging from daily to weekly sessions aimed at cognitive improvement and weight restoration. Patients were assigned to either the AN_hoop or AN_tau group. The ED clinic offers an ongoing treatment program for body image disturbances that runs in 8-week cycles and includes a maximum of 6 to 8 adult patients. This program is offered to AN patients who are within ± 4 kg of their target weight or to those recommended by their psychiatrist (i.e., psychiatrists’ clinical judgment indicates that the program will be beneficial for the patient). The treatment group consisted of AN patients with different subtypes, different levels of comorbidity, and different individual treatment programs next to the body image treatment. At the start of the 8-week cycle patients were invited to participate in the pilot after the researchers explained the procedures. All patients in a given treatment group were assigned to the same condition (either AN_hoop or AN_tau), to ensure that AN_tau patients would not be influenced by, e.g., hearing about the hoop intervention from others in their group who did participate in the hoop intervention.

HC were required to have no history of or current psychiatric disorder, substance/alcohol abuse, or medical illness (based on self-report) and a healthy BMI between 19 and 25. The latter was initially based on self-report, but height and weight were measured during the experiment by the experimenter to verify self-reported BMI. Using the EDI-II [8], HC were screened for the presence of an eating disorder.

### Materials and procedures

Body image measures were completed twice by each participant. Once in the week that body image disturbance treatment started (baseline) and once after 8 weeks following completion of body image disturbance treatment (follow-up). At the end of each TAU session (~ 75 min per session) the AN_hoop group individually completed the hoop training (~ 5–10 min per session). The AN_tau group served as a clinical control group and received identical body image disturbance treatment as the AN_hoop group, but not the hoop training. The HC group did not receive any intervention and was included to check for any natural fluctuation and/or learning effects in measures of body image over the course of 8 weeks. We Cognitive, visual, tactile, and body-scaled action aspects of body image were measured at baseline and follow-up. The tasks that were used are identical to those used in another study from our lab [7]: the *Body Attitude Test* (BAT) assessed body dissatisfaction; the *Visual Estimation Task* (VET) assessed in percentages error in estimation of shoulder, waist and hip width ; the *Tactile Estimation Task* (TET) measured in cm the distance estimation of stimuli touching the skin; the *Hoop Task* assessed in percentages the error in estimation of which hoop would exactly fit around the participant’s body.

## Results and discussion

The aim of the current research was to test in a pilot study a novel intervention for body image disturbance in AN, and to compare its effectiveness to TAU. Since this was a pilot study comparing small groups power to detect reliable effects within or between groups was low, to prevent unreliable results, no statistical analyses were performed. Instead, means were compared and any baseline to follow-up differences were interpreted based on overlapping standard errors of the mean (SEM).

**Fig. 2:**
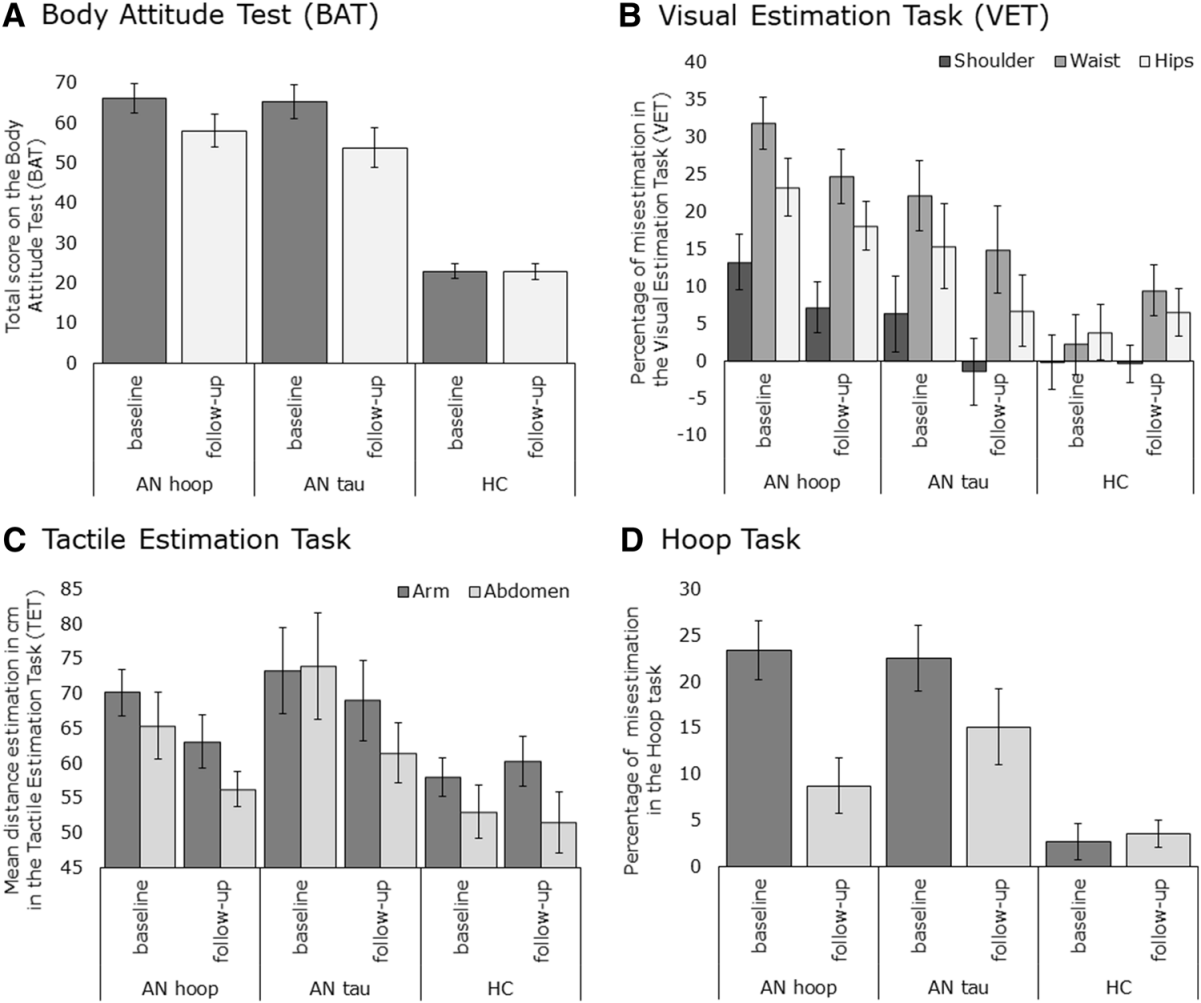
Results of body image disturbance measures at baseline and follow-up by group. **a** Cognitive measure of body image disturbance as assessed with the Body Attitude Test (BAT). Total scores are given for each participant group at baseline (before treatment started) and follow-up (following the end of treatment, after 8 weeks). **b** Visual measure of body image disturbance (BID) assessed with the Visual Estimation Task (VET). Percentage of misestimation is given for each body part and participant group at baseline and follow-up. **c** Tactile measure of BID as assessed with the Tactile Estimation Task (TET). Mean distance estimation over all trials is given for each participant group at baseline and follow-up. A score of 60 mm represents a perfect size estimate. ** d** Body-scaled action measure of BID assessed with the Hoop Task. Percentage of misestimation is given, indicating the discrepancy between the smallest hoop participants actually fitted through and the smallest hoop they judged themselves to fit through, for each participant group at baseline and follow-up

The results are presented in Fig. 2 and show that from baseline to follow-up HC remain stable on all body image disturbance measures.

AN Patients who in addition to TAU completed the 8-week hoop training performed better on several body image tasks than patients who only completed TAU for body image disturbance in both panel C and D, showing results of the tactile task and the hoop task, a decrease in size estimation from baseline to follow-up can be observed in both the AN_hoop and AN_tau group. However, from baseline to follow-up SEMs in both panel C and D do not overlap for the AN_hoop group, implying improved performance on the tasks, while SEMs do overlap for the AN_tau group, implying no improvement in performance. Thus, it was specifically found that hoop training selectively improved tactile size estimation and body-scaled action. This indicates that after completing the hoop training, patients improved in deriving body size estimates from multisensory information, such as tactile or proprioceptive signals. As hoop training explicitly focusses on experiencing body size directly by means of, e.g., moving the body, feeling with the body, it uniquely targets aspects of body image disturbance that are not addressed in TAU, which is often more cognitive in nature. Clinical experience leads to the speculation that hoop training has a large impact as it focuses on aspects of body image disturbance that are not only little addressed in TAU, but also often avoided by patients themselves. Patients frequently report a disconnection from their body (see also [9]). For them (literally) feeling their body can be a very frightening, scary, or stressful experience. Patients might generally avoid such experiences. During the hoop-training patients learn to connect with their bodily signals again and can practice with noticing, interpreting, and evaluating these signals, i.e., how does it feel when the hoop touches your body? At the same time patients are stimulated to become aware of changes in how they perceive their body. In more cognitive-directed training patients focus more on learning to recognize and change maladaptive thoughts related to ‘feeling fat’, but they do not directly address perceptual aspects of ‘feeling fat’. As such hoop training adds something new to existing interventions for body image.

Regardless of whether patients completed hoop training they also showed improvements in body attitudes, see panel A, SEMs do not overlap for both AN groups, but not in a visual body size estimation task, see panel B, SEMs overlap in both AN groups. Changes in body attitudes in both patient groups were anticipated, as both groups completed TAU, which is rather cognitive in nature. As for a lack of improvement on the visual body size estimation task, it could be that the visual estimation task that was used here was not highly compatible with the small sample size. Variation in task performance across participants was very high. Future work may benefit from including a different visual body size estimation task.

Given the small sample size of the current pilot, it is of great importance that future research is initiated, including larger populations. A limitation of the current pilot study that should be addressed in future studies is that no intervention group was included that received a different or mock body image training instead of hoop training, to compensate for extra therapy time. Furthermore, at this point it is unclear what the long term effects of the hoop training are. In this pilot only a post-treatment assessment was included, directly after completing the 8-week training. Future studies should include substantial follow-ups to get a clear understanding of the duration of the treatment effects.

Although the results of this pilot study should be interpreted with caution and require further replication in larger samples, the initial data are very promising, especially given the persistent nature of body image disturbance (BID) in AN (e.g., [3, 9, 10]). This pilot showed that hoop training, which focuses on the full spectrum of multisensory aspects of body image disturbance, is a valuable addition to TAU. What strengthens this pilot study is that a systematic investigation of body image disturbance was included, which allows for a substantial overview on which aspects are affected by the hoop training and which are not. Moreover, hoop training was specifically designed to be easily added to TAU, in terms of time management and cost effectiveness, but also (non)interference with other treatments. It should not be expected of clinicians to adopt a complete new approach in their treatment strategy, but here an elegant and short training is offered to them, that can be easily implemented and aims to facilitate treatment of body image disturbance in an patients.
